# Larger Subcortical Gray Matter Structures and Smaller Corpora Callosa at Age 5 Years in HIV Infected Children on Early ART

**DOI:** 10.3389/fnana.2017.00095

**Published:** 2017-11-02

**Authors:** Steven R. Randall, Christopher M. R. Warton, Martha J. Holmes, Mark F. Cotton, Barbara Laughton, Andre J. W. van der Kouwe, Ernesta M. Meintjes

**Affiliations:** ^1^Department of Human Biology, Faculty of Health Sciences, University of Cape Town, Cape Town, South Africa; ^2^MRC/UCT Medical Imaging Research Unit, Division of Biomedical Engineering, Department of Human Biology, Faculty of Health Sciences, University of Cape Town, Cape Town, South Africa; ^3^Children's Infectious Diseases Clinical Research Unit, Department of Paediatrics and Child Health, Tygerberg Children's Hospital & Faculty of Medicine and Health Sciences, Stellenbosch University, Cape Town, South Africa; ^4^Athinoula A. Martinos Center for Biomedical Imaging, Massachusetts General Hospital, Charlestown, MA, United States

**Keywords:** HIV/AIDS, antiretroviral, MRI, volumetric segmentation, WM, GM, pediatric

## Abstract

Sub-Saharan Africa is home to 90% of HIV infected (HIV+) children. Since the advent of antiretroviral therapy (ART), HIV/AIDS has transitioned to a chronic condition where central nervous system (CNS) damage may be ongoing. Although, most guidelines recommend early ART to reduce CNS viral reservoirs, the brain may be more vulnerable to potential neurotoxic effects of ART during the rapid development phase in the first years of life. Here we investigate differences in subcortical volumes between 5-year-old HIV+ children who received early ART (before age 18 months) and uninfected children using manual tracing of Magnetic Resonance Images. Participants included 61 Xhosa children (43 HIV+/18 uninfected, mean age = 5.4 ± 0.3 years, 25 male) from the children with HIV early antiretroviral (CHER) trial; 27 children initiated ART before 12 weeks of age (ART-Before12Wks) and 16 after 12 weeks (ART-After12Wks). Structural images were acquired on a 3T Allegra MRI in Cape Town and manually traced using MultiTracer. Volumetric group differences (HIV+ vs. uninfected; ART-Before12Wks vs. ART-After12Wks) were examined for the caudate, nucleus accumbens (NA), putamen (Pu), globus pallidus (GP), and corpus callosum (CC), as well as associations within infected children of structure volumes with age at ART initiation and CD4/CD8 as a proxy for immune health. HIV+ children had significantly larger NA and Pu volumes bilaterally and left GP volumes than controls, whilst CC was smaller. Bilateral Pu was larger in both treatment groups compared to controls, while left GP and bilateral NA were enlarged only in ART-After12Wks children. CC was smaller in both treatment groups compared to controls, and smaller in ART-After12Wks compared to ART-Before12Wks. Within infected children, delayed ART initiation was associated with larger Pu volumes, effects that remained significant when controlling for sex and duration of treatment interruption (left β = 0.447, *p* = 0.005; right β = 0.325, *p* = 0.051), and lower CD4/CD8 with larger caudates controlling for sex (left β = −0.471, *p* = 0.002; right β = −0.440, *p* = 0.003). Volumetric differences were greater in children who initiated ART after 12 weeks. Results suggest damage is ongoing despite early ART and viral load suppression; however, earlier treatment is neuroprotective.

## Introduction

Since the advent of combination antiretroviral (ARV) therapy (ART), human immunodeficiency virus (HIV) infection has become a chronic condition with ongoing damage to the body and central nervous system (CNS), especially in the developing brain of the fetus, infant and young child (van Rie et al., 2007). As CNS penetration by ART is limited, the brain may become a reservoir for the virus, with few drugs available to impact these reservoirs (van Rie et al., 2007). Substantial brain development in the first few years of life puts infected children at greater risk of neurological impairment compared to HIV infected (HIV+) adults (Tardieu et al., 2000; Mitchell, 2001). For example, language functions are more impaired in HIV+ children than in adults (van Rie et al., 2007).

To minimize effects of HIV in children, new guidelines recommend that life-long ART be initiated as soon as possible (WHO, 2013). However, some ARVs can cause neurodevelopmental impairment despite virological suppression in the CNS (Tardieu et al., 2005; van Rie et al., 2007). For example, prenatal exposure to zidovudine has been linked to mitochondrial dysfunction within the CNS (Tardieu et al., 2005), as well as chronic ARV treatment in the form of combination therapy results in abnormal BOLD response within frontal brain regions (Chang et al., 2008). Few studies have examined the long-term neurological effects of HIV infection in children in whom plasma viral loads (VL) are suppressed following early ART (before 18 months of age; Le Doaré et al., 2012; Laughton et al., 2013; Phillips et al., 2016), and very little is known about the complex nature of brain recovery following ART initiation and subsequent brain development (Tamula et al., 2003; Shanbhag et al., 2005; van Rie et al., 2007; Govender et al., 2011; Laughton et al., 2012; Whitehead et al., 2014).

Although neuroimaging can provide insights into the mechanisms underpinning neurobehavioral outcomes in HIV+ children (Hoare et al., 2014; Musielak and Fine, 2016), such studies are rare, even in developed countries, and have typically included children across wide age ranges who either started ART late (Hoare et al., 2015) or some on early ART but small numbers with VL suppression (van Arnhem et al., 2013; Sarma et al., 2014). Studies in children and youths where most (>85%) have suppressed viral loads, and some received early ART, have demonstrated lower global and local cortical and total gray matter (GM) volumes (Cohen et al., 2016; Lewis-de Los Angeles et al., 2017), white matter (WM) alterations (Ackermann et al., 2014, 2016; Andronikou et al., 2014; Uban et al., 2015), and volume reductions (Cohen et al., 2016), both subcortical volume increases and decreases and shape deformations (Lewis-de Los Angeles et al., 2016; Yadav et al., 2017), altered cortical thickness (Yadav et al., 2017), and effects of immunocompromise in infancy on basal ganglia metabolism at 5 years (Mbugua et al., 2016). These findings suggest that HIV- and/or ART-related damage may be irreversible or ongoing.

In this study we compare over a narrow age range (4.9–6.5 years of age) manually traced volumes of select subcortical structures and the corpus callosum in a unique cohort of HIV-infected children, all of whom initiated ART before 76 weeks of age and were followed since birth, to HIV-uninfected children from the same community. This is the first study using gold-standard manual tracing (Morey et al., 2009) to assess structural brain changes in perinatally acquired HIV on early ART (Hoare et al., 2014; Musielak and Fine, 2016). Amongst HIV+ children from this cohort, initiating treatment before 12 weeks showed improved cognitive performance at 11 months and improved overall health compared to starting treatment after 12 weeks (Violari et al., 2008; Laughton et al., 2012; Cotton et al., 2013). We also examined the effect of timing of treatment initiation on volume alterations. We hypothesized that HIV+ children on early treatment would experience similar perturbations observed previously in both children and adults, with neuronal thinning, resulting overall in global atrophy and loss of gray matter and white matter volume. In addition, we hypothesized that earlier treatment would mitigate the effects of HIV and accompanying brain damage leading to smaller reductions in volume in children initiating ART earlier.

## Materials and methods

This study included 43 HIV+ Xhosa children from the randomized CHER (Children with HIV early antiretroviral) trial in follow-up at the Children's Infectious Diseases Clinical Research Unit, Tygerberg Children's Hospital, Cape Town (Laughton et al., 2012). As part of the CHER trial, infants with CD4 percentage (CD4%) of at least 25% were randomized to one of the following three treatment arms: ART-Def (ART Deferred until CD4% < 25% in first year or CD4% < 20% thereafter, or if clinical disease progression criteria presented); ART-40W (ART initiated before 12 weeks of age and interrupted after 40 weeks); and ART-96W (ART initiated before 12 weeks of age and interrupted after 96 weeks). ART was restarted in ART-40W and ART-96W for CD4% decline or clinical evidence of disease progression. (Violari et al., 2008; Cotton et al., 2013). One child not adhering to ART was excluded. Since some children in the ART-Def arm met criteria for almost immediate initiation of ART, the children were grouped here based on age at treatment initiation, specifically those who received ART at or before 12 weeks (ART-Before12wks) and those who received treatment after 12 weeks (ART-After12wks). Of the 27 who received ART before 12 weeks, 9 remained on continuous ART in line with clinical criteria governing interruption. Treatment was interrupted in 18, two of whom had not met ART restart criteria at the time of scan as they remained clinically well with CD4% of at least 20%.

Eighteen HIV uninfected Xhosa children were recruited from an interlinked vaccine trial as controls (Madhi et al., 2010). They comprised 12 HIV exposed uninfected (HEU) children born to HIV+ mothers but who tested HIV negative (PCR) at baseline and 30 days after the third dose of vaccine, and 6 HIV unexposed and uninfected children (HU) born to HIV seronegative mothers (tested after 24 weeks gestation) who remained seronegative at enrolment.

All children received MRI scanning on a 3T Allegra MRI (Siemens, Erlangen, Germany) at the Cape Universities Brain Imaging Centre (CUBIC) according to protocols approved by the Human Research Ethics Committees of the Universities of Cape Town and Stellenbosch. All parents provided written informed consent and all children provided oral assent. High-resolution T1 weighted images were acquired using a volumetric echo-planar imaging (EPI) navigated (Tisdall et al., 2012) multi echo magnetization prepared rapid gradient echo (MEMPRAGE) sequence (van der Kouwe et al., 2008). Imaging parameters were: FOV: 224 × 224 mm^2^; 144 sagittal slices, TR: 2,530 ms; TE: 1.53/3.19/4.86/6.53 ms; TI: 1,160 ms; Flip angle: 7°; voxel size: 1.3 × 1.0 × 1.0 mm^3^, scan time 5:20 min. The 3D EPI navigator provided real-time motion tracking and correction. BrainVoyager QX software (Brain Innovation, Maastricht) was used to transform DICOM files into the AC-PC plane and rotate images for hemispherical symmetry.

**Figure 1:**
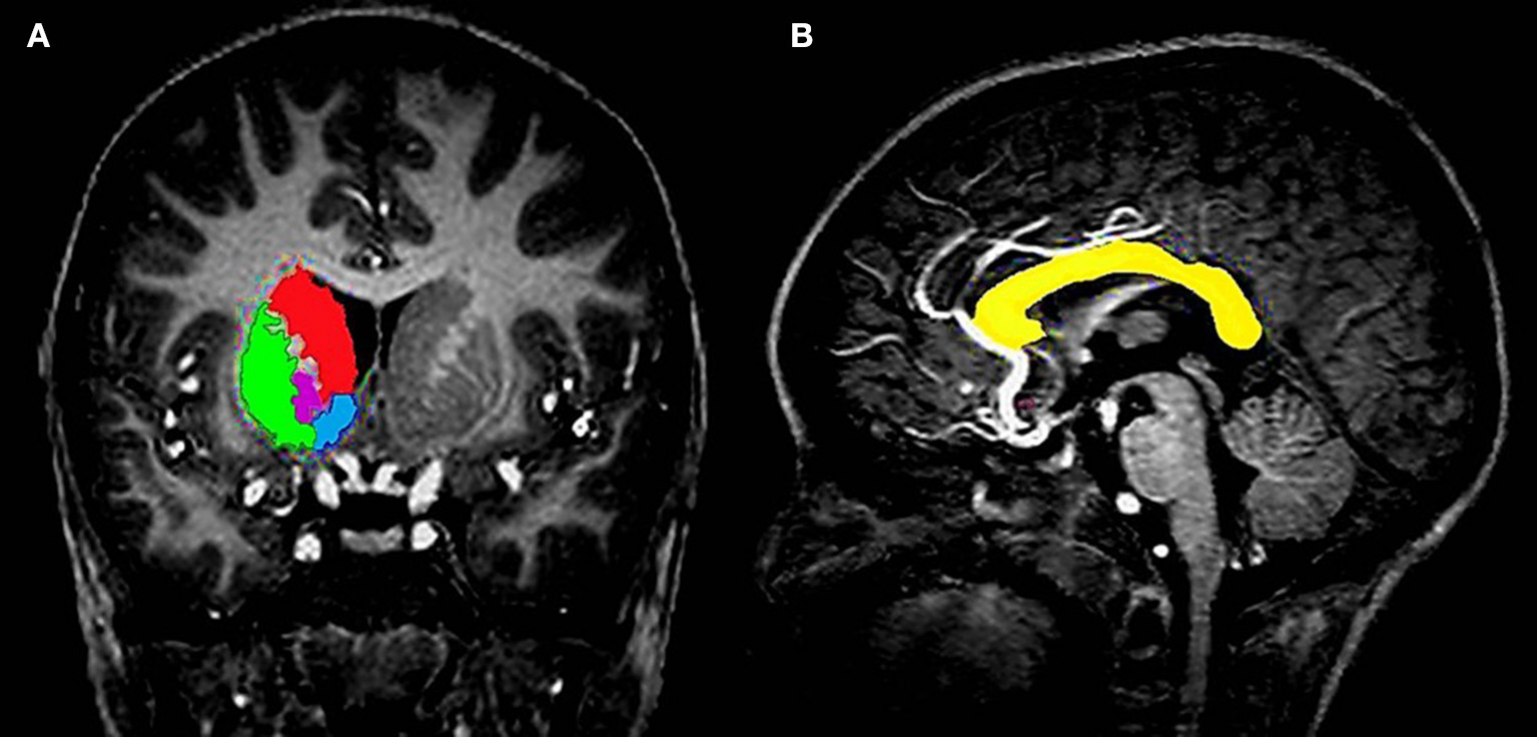
**(A)** Coronal view showing the four different structures that were manually segmented in the basal ganglia, namely the caudate (red), putamen (green), globus pallidus (magenta), and nucleus accumbens (blue). **(B)** Sagittal view showing a tracing of the corpus callosum in a midline slice (yellow).

Due to its frequency in the HIV literature, the basal ganglia (Figure 1A) was the primary subcortical region of interest (ROI), with the corpus callosum (Figure 1B) functioning as the white matter analog. All gray matter structures within the basal ganglia were manually segmented, with the exception of the substantia nigra and subthalamic nuclei. The structures included the caudate head, nucleus accumbens (NA), globus pallidus (GP), and putamen (Pu). All structures were manually traced using MultiTracer software (http://bishopw.loni.ucla.edu/MultiTracer/) on a Lenovo ThinkPad X200 tablet and integrated active digitizer stylus.

All tracings were performed at 4X magnification as further magnification resulted in visible pixelation of the images. Image files were scaled according to the prescribed voxel intensity range (Woods, 2003); the contrast used in this study ranged from Pixel Intensity Units of 13,000 to 18,000 dependent on the structure. Screen brightness for the tracing tablet was set to 100% and brightness of images was set to 1.34 (49% of a maximum unadjusted default scale value of 2.72) for most structures (Woods, 2003).

The contours of selected brain structures were completed by manually outlining the structures on MR images, slice by slice. For standardization purposes, tracing of all subcortical gray matter structures were performed in the coronal plane. Based on this tracing, we present the frust volumes output by MultiTracer. The expert neuroanatomist (SR) who performed all tracings was blinded to all participant data. Inter-rater and intra-rater reliabilities were assessed for each structure using independent measurements for 10 participants who were randomly selected by another expert neuroanatomist (CW) and the primary tracer (SR), respectively, and assessed via Pearson and intra-class correlation.

Data and statistical analyses were performed in IBM SPSS Statistics 23. ANOVA was used to compare structure volumes of uninfected children to infected children, and to children who initiated ART before and after 12 weeks. Additionally, amongst infected children, we used linear regression to examine associations of structure volumes with timing of ART initiation. Since treatment was interrupted in a subset of the children who received ART before 12 weeks and interruption is characterized by a subsequent drop in CD4 cell count and increased VL, which may negate the potential benefits of earlier ART, multiple regression was used to control for duration of interruption. Duration of interruption was set to zero for children in whom treatment was not interrupted. Sex was controlled for in all analyses as it affects brain development (Gur et al., 1999; Gilmore et al., 2007). Finally, to explore whether volume alterations may be due to an inflammatory response, we examined relationships between structure volumes and CD4/CD8 ratio as a proxy for immune health, both at enrolment and at scan. Due to the fact that statistical analyses were repeated for 9 anatomical structures, we indicate for each test which results survive Bonferroni correction (*p* < 0.0056).

Since lower total GM and WM volumes have been reported in HIV+ youths (Cohen et al., 2016; Lewis-de Los Angeles et al., 2017), total intracranial volumes (ICVs) were derived for all children using FreeSurfer (Fischl, 2012) and considered a potential confounder. Additional control variables considered included age at scan and birthweight. Control variables showing group differences were controlled for using analysis of covariance (ANCOVA) and multiple regression.

## Results

We present data for 61 Xhosa children (mean age ± s.e. = 5.4 ± 0.04; 25 male). Of 18 uninfected controls, 12 were HEU and 6 HU. Of the 43 infected children, 27 received ART before 12 weeks, and 16 after. Sample characteristics are summarized in Table 1. Since uninfected children were slightly older than infected children, and children who received ART before 12 weeks had smaller ICVs than uninfected controls, we controlled for ICV and age at scan in all analyses involving uninfected and infected children. While no children had suppressed VLs at enrolment, VL was suppressed in 93% of infected children at time of scanning. Children receiving ART before and after 12 weeks did not differ on any of the clinical variables. No significant effect of interruption on structure volume was observed, nor did duration of interruption show any relation with structure volumes (all *p*'s > 0.18).

**Table 1:** Sample characteristics.

**Biographical Data**		**Uninfected Controls (*n* = 18)**	**HIV infected (*****n*** = **43)**			***F/χ^2^***	***p***
			**Total Infected (*n* = 43)**	**ART-Before12Wks (*n* = 27)**	**ART-After12Wks (*n* = 16)**		
Sex: Male	*n* (%)	8 (44)	17 (40)	11 (41)	6 (38)	0.17	0.918
Age: Scan [Yrs]^a^	Mean (*SD*)	5.6 (0.50)	5.4 (0.25)	5.4 (0.20)	5.3 (0.30)	4.61	0.014
	(Range)	(5.1–6.5)	(4.9–5.9)	(5.0–5.9)	(4.9–5.9)		
Birth weight [g]	Mean (*SD*)	3,105 (642)	3,104 (400)	3,094 (426)	3,122 (363)	0.02	0.983
ICV [mm^3^]^b^	Mean	1.36 × 10^6^	1.27 × 10^6^	1.26 × 10^6^	1.31 × 10^6^	5.05	0.009
	(*SD*)	(0.11 × 10^6^)	(0.11 × 10^6^)	(0.12 × 10^6^)	(0.08 × 10^6^)		
**HIV-INFECTED CLINICAL DATA**							
**Treatment**							
Cumulative Treatment [Wks]	Median (IQR)		240.9 (39.0)	236.3 (47.9)	241.4 (24.0)	0.12	0.728
Age at ART initiation [Wks]	Mean (*SD*)		18.3 (16.4)	8.3 (1.6)	35.7 (16.0)	78.78	<0.001
	(Range)		(6.5–75.5)	(6.5–12.0)	(18.37–75.5)		
Age VL Suppression [Wks]	Median (IQR)		43.1 (41.4)	33.5 (16.0)	61.9 (60.0)	9.71	0.002
	(Range)		(29.0–161.0)	(30.5–101.0)	(29.0–161.0)		
Number ART Interrupted	(*n*)		18	18	0		
Age at Interruption [Wks]^c^	Median (IQR)		50.8 (54.8)	50.8 (54.8)	Not Interrupted		
	(Range)		(46.6–114.6)	(46.6–114.6)			
Length of Interruption [Wks]^c^	Median (IQR)		45.8 (74.4)	45.8 (74.4)			
	(Range)		(11.0–208.1)	(11.0–208.1)			
**ENROLMENT^α^**							
CD4%	Mean (*SD*)		36 (8)	35 (8)	37 (7)	0.44	0.511
CD4/CD8 Ratio	Mean (*SD*)		1.44 (0.75)	1.42 (0.80)	1.45 (0.64)	0.06	0.813
CD4 Count (Cells/mL)	Median (IQR)		1,836 (1,219)	1,835 (1,380)	1,889 (799)	0.23	0.633
CD8 Count (Cells/mL)	Median (IQR)		1,398 (1,364)	1,493 (1,191)	1,387 (1,543)	0.04	0.844
Plasma Viral Load (RNA/ml)			Total				
High (≥750001)	*n* (%)		23 (53)	12 (44)	11 (61)	2.39	0.122
Low (400–750000)	*n* (%)		20 (47)	15 (56)	5 (39)		
Suppressed (≤399)	*n* (%)		0 (0)	0 (0)	0 (0)		
**PRESCAN**							
Sample to Scan (Days)	Median (IQR)		28 (51.5)	28 (59.6)	33 (60.5)	0.567	0.327
	(Range)		(0–223)	(0–223)	(0–144)		
CD4%	Mean (*SD*)		37 (8.00)	35 (8)	37 (7)	1.24	0.271
CD4/CD8 Ratio	Mean (*SD*)		1.32 (0.65)	1.32 (0.65)	1.33 (0.48)	0.15	0.702
CD4 Count (Cells/mL)	Median (IQR)		1,029 (677)	1,029 (677)	931 (620)	0.02	0.880
CD8 Count (Cells/mL)	Median (IQR)		1,011 (582)	878 (299)	863 (778)	0.02	0.900
Plasma Viral Load (RNA/ml)^§^			Total				
High (≥750001)	*n* (%)		1 (2)	0 (0)	1 (6)	1.95	0.378
Low (400–750000)	*n* (%)		4 (9)	3 (11)	1 (6)		
Suppressed (≤399)	*n* (%)		38 (89)	24 (89)	14 (88)		

*Values are Mean (SD), Median (IQR), number (%)*.

*χ2 from Kruskal–Wallis ANOVA or categorical Associations; F statistic from Univariate ANOVA for parametric data between treatment groups and controls. ICV, Intracranial Volume; Yrs, Years; Wks, Weeks; g, Grams; mm, millimeters; SD, Standard deviation; IQR, Interquartile Range*.

a *ART-Before12 wks, ART-After12 wks < Uninfected Controls (both p's < 0.05)*.

b *ART-Before12 wks < Uninfected Controls (p < 0.01)*.

c *Only including 18 children from the ART-Before12 wks group who were interrupted; 2 had not yet restarted ART at time of scanning*.

α *CD8 count at enrolment data missing for 1 ART-After12 wks girl*.

§ *Plasma Viral Load Chi Tests had fewer than 5 cases*.

All inter-rater Pearson correlations for manually traced volumes were significant (all *p*'s < 0.05) and ranged from *r* = 0.71 for the left caudate to *r* = 0.93 for the corpus callosum. Cronbach's α's were above 0.8 in all regions. Intra-rater Pearson correlations were greater than *r* = 0.82 (all *p'*s < 0.001), and Cronbach's α's were all above 0.83.

**Figure 2:**
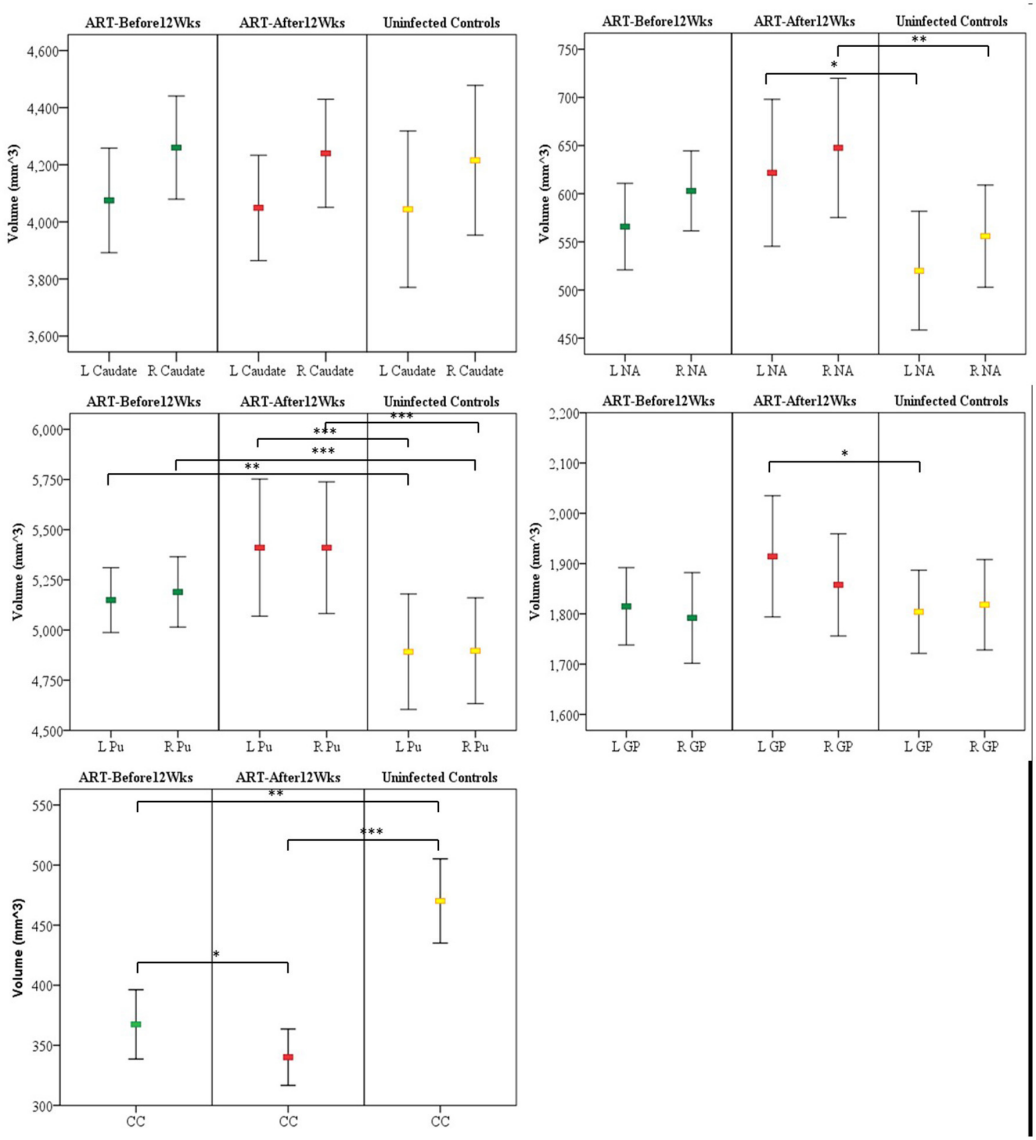
Mean and confidence interval of structure volumes in children who initiated ART before and after 12 weeks and uninfected controls. Volumes were compared using ANCOVA adjusted for sex, intracranial volume, and age at scan. **(A)** Caudate **(B)** nucleus accumbens (NA) **(C)** Putamen (Pu) **(D)** Globus pallidus (GP) **(E)** Corpus callosum (CC) (L, left; R, right); ^*^p < 0.05, ^**^p < 0.01, ^***^p < 0.001.

In Table 2 we compare structure volumes between HIV+ and uninfected children, and in Figure 2 between uninfected children and children who received ART before and after 12 weeks, controlling for sex, ICV, and age at scan. Infected children showed gray matter (GM) volume increases in left (L) NA, albeit below conventional levels of significance, right (R) NA, bilaterally in Pu, and L GP, effects that appear to be largely attributable to volume increases in children who initiated ART after 12 weeks. In contrast to GM volume increases, CC was smaller in infected children with reductions evident both in children initiating ART before and after 12 weeks. Only in CC did volumes differ significantly between children initiating ART before and after 12 weeks, with the latter showing greater volume reductions compared to uninfected controls than children who initiated ART before 12 weeks. HIV-related volume increases in the Pu and reductions in the CC remain significant after Bonferroni correction for multiple comparisons.

**Table 2:** Comparison of structure volumes between HIV infected and uninfected children.

**ROI (mm^3^)**	**Uninfected Controls (*n* = 18)**		**HIV infected (*n* = 43)**		***F***	***p***
	**Mean**	**(*SD*)**	**Mean**	**(*SD*)**		
L Caudate	4,045	(551)	4,065	(419)	0.312	0.58
R Caudate	4,216	(527)	4,253	(418)	0.904	0.35
L NA	520	(124)	587	(127)	3.587	0.06
R NA	556	(107)	620	(118)	5.205	0.03
CC	470	(71)	357	(65)	19.216	<0.001
L Pu	4,892	(578)	5,246	(516)	15.768	<0.001
R Pu	4,897	(530)	5,272	(518)	18.097	<0.001
L GP	1,804	(166)	1,852	(210)	3.907	0.05
R GP	1,818	(181)	1,816	(215)	2.016	0.16

*F-value from Analysis of Covariance adjusted for sex, intracranial volume and age at scan*.

**Figure 3:**
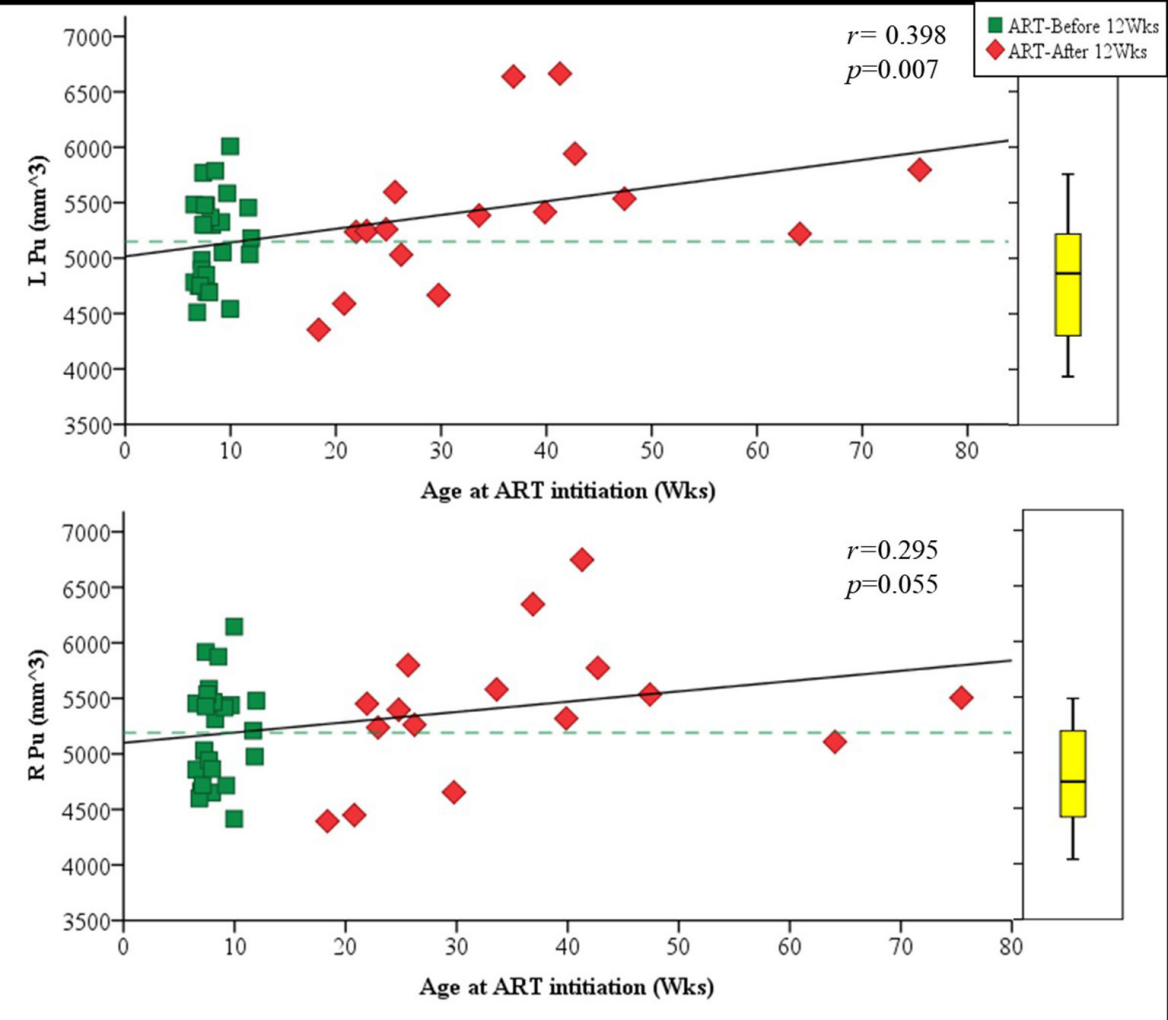
Plots showing the association of volume in the left **(Top)** and right **(Bottom)** putamen with age of ART initiation. The box plot on the right indicates the median volume and IQR for uninfected controls in the same age range as the infected children.

**Figure 4:**
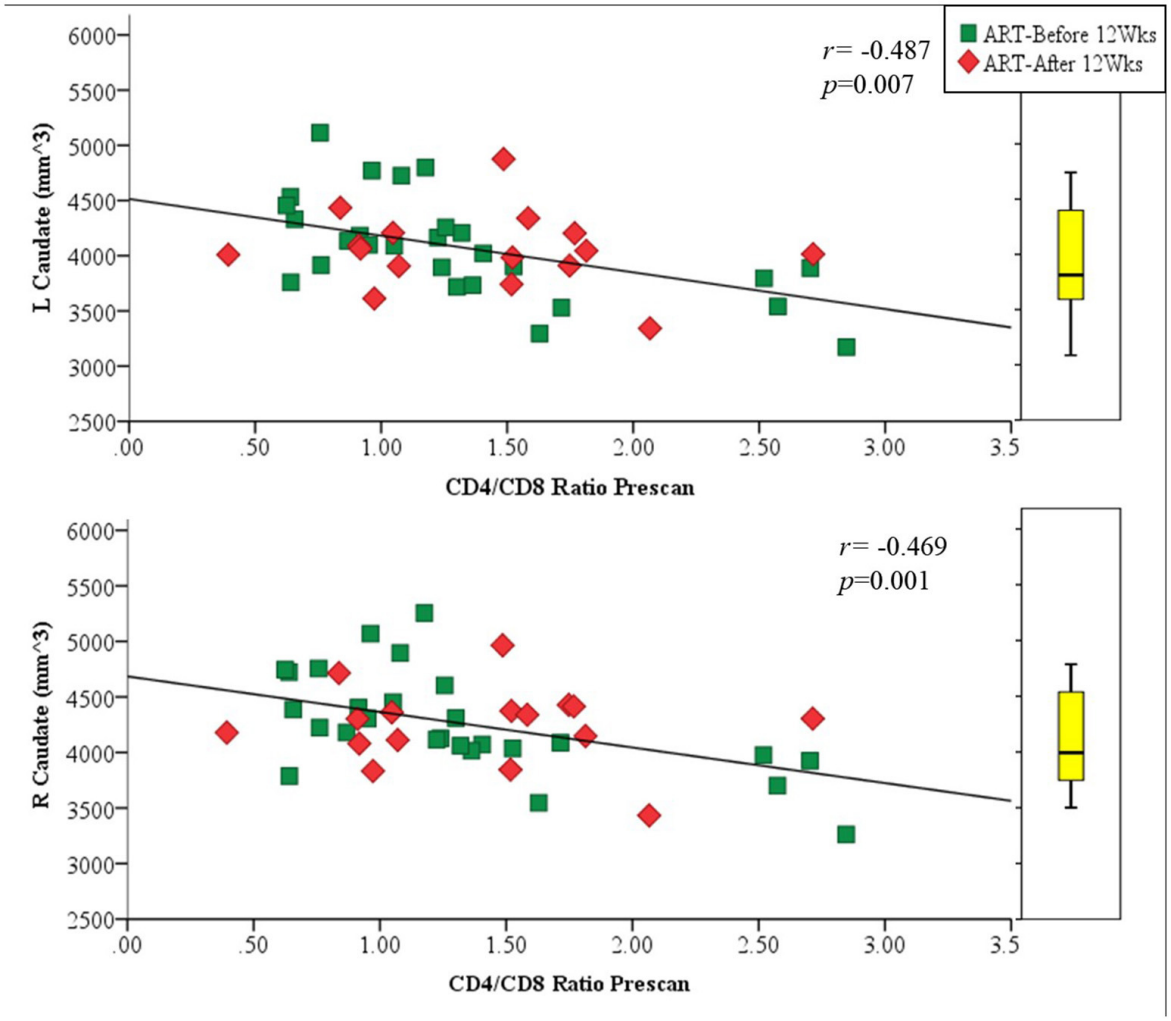
Association of left **(Top)** and right **(Bottom)** caudal volumes with CD4/CD8 ratio at scan as a proxy for immune health. The box plot indicates the distribution of caudate volumes in healthy uninfected controls, excluding the 6 children older than 6 years to match the age range of the uninfected children.

Amongst infected children, increasing age of ART initiation was associated with larger Pu volumes bilaterally (Figure 3, Table 3), with the association on the left surviving Bonferroni correction for multiple comparisons. Although caudate volumes did not show any group differences or relation with timing of ART initiation, amongst infected children, lower CD4/CD8 ratio at the time of scanning was associated with caudate volume increases bilaterally (left *r* = −0.487, *p* = 0.007; right *r* = −0.469, *p* = 0.001; Figure 4), effects that remained significant when controlling for sex (left β = −0.471, *p* = 0.002; right β = −0.440, *p* = 0.003) and adjusting for multiple comparisons. Lower enrolment CD4/CD8 was also weakly associated with greater caudal volumes at age 5 years (left *r* = −0.252 *p* = 0.107; right *r* = −0.298 *p* = 0.056), although these relations did not survive when controlling for sex (left β = −0.217, *p* = 0.201; right β = −0.226, *p* = 0.172). No other regions showed association between volumes and immune health at enrolment (all *p*'s > 0.2) or at scan (all *p*'s > 0.5).

**Table 3:** Associations in infected children of structure volume with age at ART initiation.

**ROI**	**Association with Age at ART Initiation (*n* = 43)**					
	***r***	***p***	**β_1_**	***p***	**β_2_**	***p***
L Caudate	0.071	0.651	0.060	0.701	0.193	0.230
R Caudate	0.150	0.336	0.134	0.380	0.227	0.161
L NA	0.272	0.078	0.269	0.085	0.296	0.080
R NA	0.230	0.138	0.235	0.133	0.286	0.091
CC	−0.030	0.847	−0.038	0.808	0.016	0.924
L Pu	0.398	0.007	0.385	0.010	0.447	0.005
R Pu	0.295	0.055	0.288	0.063	0.325	0.051
L GP	0.256	0.098	0.240	0.113	0.233	0.155
R GP	0.209	0.178	0.193	0.204	0.264	0.104

*r is the Pearson correlation coefficient; β1 is the standardized regression coefficient adjusted for sex; β2 is the standardized regression coefficient adjusted for sex and duration of interruption*.

## Discussion

This is the first study to examine over a narrow age range the impact of HIV on subcortical gray matter and corpus callosum volumes in a cohort of children receiving ART before 18 months of age and VL suppression by 109 weeks in all but three children. In contrast to most previous studies in HIV+ adults and children that reported gray matter atrophy (Tardieu et al., 2000; Crain et al., 2010; Becker et al., 2011; Chang et al., 2011; Cohen et al., 2016; Musielak and Fine, 2016; Lewis-de Los Angeles et al., 2017), we found HIV-related subcortical GM volume increases at 5 years in the left GP and bilaterally in the NA and Pu, but CC reductions. Association of increasing age of ART initiation with greater volumes in the Pu suggest that treatment timing plays a role in these observed volume increases. The fact that both GM and WM volume differences were largest in children who initiated ART after 12 weeks point to greater protection from earlier treatment, which is consistent with the findings from other studies in this cohort (Laughton et al., 2012; Mbugua et al., 2016). The fact that associations of Pu and NA volumes with treatment timing strengthened after adjustment for interruption supports findings from a previous DTI study in the same cohort that found the lowest fractional anisotropy in a region in the corticospinal tract in treatment-interrupted children suggesting that ART interruption may negate the benefit of earlier ART (Ackermann et al., 2016).

Even though most studies in children find cortical and total gray matter atrophy in HIV infection (Cohen et al., 2016; Lewis-de Los Angeles et al., 2017), regional GM volume increases have been reported (Sarma et al., 2014). Although, few studies have examined subcortical volumes specifically, two recent studies reported HIV-related regional subcortical GM increases. Blokhuis et al. (2017) found trend-level Pu increases in HIV+ children, and Yadav and colleagues larger nucleus accumbens and smaller hippocampi (Yadav et al., 2017). The authors postulated inflammatory processes and chronic stress as possible explanations for the volume increases. Putamen hypertrophy in HIV+ adults has also been attributed to possible inflammation and dopaminergic system dysfunction (Castelo et al., 2007). The absence of association of CD4/CD8 ratio, as a marker of immune health, with volumes in regions showing HIV-related increases in our study, suggest that observed volume increases are not necessarily due to inflammatory processes in these specific regions.

Compared to the relative abundance of literature attributing structural atrophy to gliosis and inflammation (Bates et al., 2002; Miller et al., 2002; Mehta et al., 2003; Pekny and Pekna, 2016; Pérez-Cerdá et al., 2016), the mechanisms for pathology-induced hypertrophy of brain structures are not well-understood. Although enlargement of the striatum has been associated with dopaminergic system dysfunction in recreational and antipsychotic drug use (Selemon et al., 1999; Jacobsen et al., 2001; Dazzan et al., 2005; Jernigan et al., 2005), and linked specifically to elevated glial densities in rhesus monkeys administered antipsychotic drugs (Selemon et al., 1999), smaller volume differences in children who initiated ART earlier in our study suggests that the gray matter hypertrophy observed here is not due to gliosis resulting from ART drug interactions. Notably, gray matter hypertrophy has been observed in patients with obstructive sleep apnea (Rosenzweig et al., 2013). Since short-term hypoxic events have been shown to induce reactive gliosis and neuronal death in rats (Aviles-Reyes et al., 2010), ischaemic preconditioning may cause long-lasting neuronal changes within the brain. This may be relevant here as cerebrovascular alterations have been reported in HIV+ children (Shah et al., 1996; Patsalides et al., 2002). Increased metabolic demand in the presence of reactive gliosis (Epstein and Gelbard, 1999; Walsh et al., 2004) and mitochondrial dysfunction in HIV+ children (Tardieu et al., 2005; Crain et al., 2010; Takemoto et al., 2017) may create localized subclinical ischaemic/hypoxic stress within the CNS. Angiogenesis from ischaemic preconditioning may be exacerbated by the *HIV-Tat* gene, a heparin-binding angiogenic growth factor (Das et al., 2016), expressed by infected cells. The exact mechanisms of gray matter hypertrophy in HIV+ children requires further investigation.

Volumetric MRI studies in HIV have mostly relied on automated/semi-automated techniques (Hoare et al., 2014; Sarma et al., 2014; Musielak and Fine, 2016), which typically involve some form of co-registration to a template based on an adult brain, using contrast or other feature discriminators. These techniques may be suboptimal for diagnoses where regional or global brain volumes may be affected, or when examining children. It has been shown that using age-matched pediatric brain templates in pediatric studies lead to considerably different tissue distribution from that obtained with an adult-based template (Yoon et al., 2009). This may explain why previous studies of subcortical volumes using FreeSurfer either failed to detect group differences (Lewis-de Los Angeles et al., 2016), or detected fewer and less significant differences (Blokhuis et al., 2017; Yadav et al., 2017) than in our study. Using FreeSurfer automated segmentation in our cohort, within the gray and white matter structures investigated, there were no significant volumetric differences between groups, apart from the left globus pallidus (GP) which was smaller in infected children (L GP: Mean (*SD*): HIV 1,775 ± 192 mm^3^, controls 1,949 ± 255 mm^3^, *F* = 7.848, *p* = 0.007), and the CC that tended to be larger (HIV 469 ± 89 mm^3^, controls 431 ± 68 mm^3^, *F* = 2.871, *p* = 0.096). In both structures, the findings were opposite to those generated using manual segmentation, indicating that automated methods may be inappropriate for pediatric populations, especially in the presence of pathology, and that manual segmentation may be more sensitive to detect subtle changes.

The highest concentration of HIV is observed in the caudate nuclei and CC (Thompson et al., 2006; Ances et al., 2012), possibly due to their proximity to the ventricles. The proximity of these structures to the ventricles, and thus CSF, allows for easier penetration of HIV-infected mononuclear cells permitting higher concentrations of HIV toxins in these sites (Oster et al., 1993; McClernon et al., 2001; Thompson et al., 2006; Kumar et al., 2007; Ances et al., 2012; Andronikou et al., 2014). This would in turn also apply to certain ARVs that more readily permeate through the blood-brain-barrier (BBB) and pass within the CSF to these sites. As such, location may explain the association seen in the left and right caudate with CD4/CD8 ratio at the time of scanning as a measure of immune health in which the more immunocompromised children at the time of scan had larger caudate volumes bilaterally. This relationship, which survives correction for multiple comparisons, suggests that at this age the caudate is particularly susceptible to the concurrent immune state.

Due to their greater distance from the ventricles, the Pu and NA may be less readily penetrated by the HIV-infected mononuclear cells. However, higher cerebral blood flow of these areas have been observed in HIV+ children (Blokhuis et al., 2017). Another hypothesis may be that once HIV invades the area and viral infection spreads, removal of metabolic waste and virions may be slower in these structures compared to the caudate. This may explain the larger Pu and NA observed in HIV+ children. Thus, presence of inflammation in the form of reactive gliosis and leukocyte infiltration, resulting in slightly increased structure size, may be because of prolonged HIV exposure in the Pu and NA rather than a present acute, transient, and compromised immunological state, as in the left and right caudate.

Our finding of reduced CC volume in HIV infection is consistent with that of most previous studies (Thompson et al., 2006; Chiang et al., 2007; Hasan et al., 2009; Dewey et al., 2010; Heaps et al., 2012; Hoare et al., 2012; Sarma et al., 2014; Yadav et al., 2017), although CC volume and thickness were similar to those in controls in a study by Andronikou et al. (2015) including infected children from the same cohort studied here. In our study, the largest volume difference between infected and uninfected children was observed in the CC, with the CC of HIV+ children being on average 24% smaller at 5 years of age than in their uninfected counterparts. Thompson et al. (2006), who similarly found a 25% decrease in the thickness of the CC in HIV+ adults compared to uninfected controls (Thompson et al., 2006), postulated that the overall thickness of the CC is impacted by the loss of peripheral WM. It has been suggested that callosal thickness and volume may be used as biomarkers of overall global WM integrity (Thompson et al., 2006; Andronikou et al., 2014). However, DTI in children from the same cohort studied here revealed no differences in WM integrity between infected and uninfected children in the CC at 5 years of age (Ackermann et al., 2016), despite volume reductions. Since DTI analyses involve co-registration to a template before performing voxel wise statistical comparisons, DTI outcomes would not be impacted by volume differences. As such, making inferences regarding microstructural integrity from macrostructural data may be inappropriate.

The clinical manifestations of HIV infection in these children do not paint a clear picture of causation. VL was suppressed (≤399 RNA/ml) in 93% of children at time of scanning. A previous study in 128 perinatally infected children, who were born prior to the adoption of preventative treatment or ART guidelines, found that 21% showed characteristic evidence of HIV infection induced encephalopathy, despite at least 74% of these children having VL suppression at the time of diagnosis (Cooper et al., 1998). Findings of VL suppression in plasma may, however, not be representative of the compartmentalized and unique viral reservoirs in the CNS (Strain et al., 2005; Pillai et al., 2006). Further, there could be ongoing effects from damage or delayed development arising during the initial phases of HIV invasion, prior to ART initiation, which may have occurred *in utero* in some participants. In our study, all HIV+ treatment groups had elevated VL at study entry (7 weeks of age) thus possibly facilitating HIV entry into the CNS early on (Ivey et al., 2009). Neuroinvasion by HIV can occur as early as the initial 10 days post-infection (Lackner et al., 1994). Early ART administered at around 8.4 ± 1.6 weeks of age is perhaps too late for individuals already experiencing high viral loads. Damage to the basal ganglia, and thus corpus striatum is dictated by the rate of initial insult (Brouwers et al., 2000; Becker et al., 2011). This may be evidenced by our observation of CD4/CD8 ratio at enrolment showing weak trend-like association with the size of the left and right caudate. This ties in with spectroscopy findings involving the same cohort at the same age, where basal ganglia metabolite levels (choline, NAA) were associated with CD4/CD8 at enrolment, suggesting that early infiltration and damage caused by HIV persists into early childhood (Mbugua et al., 2016). A confounding issue here may be the duration of ART itself. A trend of duration of ART with prefrontal CC thickness was observed in this cohort previously (Ackermann et al., 2014; Andronikou et al., 2014). It has also been shown that ART decreases motor and working memory performance in both children and adults (von Giesen et al., 2003; Chang et al., 2008; Laughton et al., 2012) which suggests that there may be some complicated interplay between the benefits and risks associated with early ART.

A limitation to the study is the inclusion of total ICV calculated using FreeSurfer automated methods. Intrinsically, using an automated measure to standardize manually derived volumes may add to inaccuracies (Morey et al., 2009; Yoon et al., 2009; Dewey et al., 2010; Narayanan et al., 2016). However, our results do not change significantly without the inclusion of ICV. In addition, a limitation in sampling bias may exist. As the HIV+ Xhosa children formed part of a longitudinal cohort followed since birth with regular follow-up visits to the clinic, it is possible that they are at an added advantage as they could be receiving better health care than the normal standard community care, despite standardized treatment as per the current WHO guidelines. These children also received neuropsychological and behavioral testing giving them additional opportunities for individual case specific and HIV-related support. Children who maintained participation may also have nuanced home environments which allowed them to continue longitudinally.

Overall findings of this study suggest that perinatal HIV infection targets select structures of the basal ganglia, and that these effects are observable at 5 years of age despite early ART and VL suppression. In HIV+ children, both the NA and Pu are enlarged bilaterally, as well as the left GP. In contrast, the CC is smaller compared to uninfected children. Our results suggest that earlier treatment is neuroprotective. Early therapy before severe viral replication would be advantageous, as evidenced by increased structure size of the bilateral Pu with increased delay to initiate ART.

## Author contributions

SR, CW, and EM designed this study and developed the methodology. SR, MH, and EM drafted the manuscript. EM, AvdK, and BL are Principal Investigators. AvdK provided Technical expertise and data processing. SR conducted manual segmentation and performed statistical analyses. CW trained SR in manual segmentation protocol and conducted initial inter-rater reliabilities. MC and BL offered clinical expertise, and monitored and collected clinical data of participants longitudinally. All authors contributed to the manuscript and revised it critically.

### Conflict of interest statement

The authors declare that the research was conducted in the absence of any commercial or financial relationships that could be construed as a potential conflict of interest.
